# Challenges in diagnosis and treatment of long COVID

**DOI:** 10.3389/fmed.2025.1641411

**Published:** 2025-08-21

**Authors:** Lei Gu, Jian Yue, Jing Lin, Zeyi Liu, Jian-an Huang

**Affiliations:** ^1^Department of Pulmonary and Critical Care Medicine, The First Affiliated Hospital of Soochow University, Suzhou, China; ^2^Institute of Respiratory Diseases, Soochow University, Suzhou, China; ^3^Suzhou Key Laboratory for Respiratory Diseases, Suzhou, China; ^4^The People’s Hospital of Gaozhou, Gaozhou, China; ^5^Department of Infectious Diseases, The First Affiliated Hospital of Soochow University, Suzhou, China

**Keywords:** long COVID, post-COVID condition, SARS-CoV-2, diagnosis, treatment

## Abstract

Despite a large population affected by COVID-19, awareness of long COVID among clinicians is surprisingly limited. This has led to delayed or missed diagnoses and, consequently, inappropriate treatments that fail to address patients’ specific needs. Our study highlights the gaps in knowledge and the barriers to effective management of long COVID in the healthcare system. Through this work, we aim to bring attention to these critical issues and advocate for urgent action to improve the clinical management of long COVID.

Long COVID, also known as post-COVID condition, which defined by WHO as the persistence of symptoms for at least 2 months, typically beginning within 3 months of a confirmed or probable SARS-CoV-2 infection, which cannot be explained by another condition, has emerged as a significant public health concern worldwide (1). A large-scale survey by Qin et al. (2) found that approximately 10–30% of Chinese participants experienced persistent symptoms after SARS-CoV-2 infection, including fatigue, memory decline, reduced exercise capacity, cough or expectoration, thirst, sleep disturbances, and brain fog. Additionally, a systematic review and meta-analysis estimated that nearly 50% of COVID-19 patients in China may suffer from long COVID (3). A synthesis of 144 studies estimated a global pooled prevalence of long COVID at 36%, with regional variation observed: Asia, 35%; Europe, 39%; North America, 30%; and South America, 51% (4). Another meta-analysis of 16 studies reported a higher global pooled prevalence of 41.79%, with regional estimates ranging from 46.28% in Europe, 46.29% in America, 49.79% in Asia, and 42.41% in Australia (5). However, existing studies tend to emphasize prevalence rather than the pressing challenges of early identification, timely diagnosis, and clinical management.

There is currently no unified, internationally agreed diagnostic standard for long COVID, which presents challenges for clinicians. Long COVID was defined as a chronic condition present ≥3 months after SARS-CoV-2 infection according to Centers for Disease Control and Prevention (CDC) (6). NICE uses long COVID to identify and diagnose both ongoing symptomatic COVID-19 (from 4 to 12 weeks) and post-COVID-19 syndrome (12 weeks or more) (7). The NASEM defined long COVID as an SARS-CoV-2 infection-associated chronic condition present ≥3 months, with relapsing/remitting or progressive course (8). Discrepant definitions across major bodies (e.g., WHO, CDC, NICE, and NASEM) lead to heterogeneous case ascertainment and are a key reason why the condition remains challenging to diagnose in practice.

Currently, long COVID diagnosis primarily relies on patient-reported symptoms and the physician’s subjective interpretation. Many hallmark symptoms—such as fatigue, brain fog, and memory decline—lack objective biomarkers, leading to inconsistent diagnoses and under-recognition in clinical settings. Moreover, long COVID symptoms often overlap with those of other chronic illnesses, including psychiatric disorders, autoimmune conditions, and post-infectious syndromes, further complicating diagnosis. Menezes et al. (9) identified 212 differentially expressed genes in individuals with long COVID by blood transcriptomics, and found that antisense ORF1ab RNA and FYN RNA emerged as independent diagnostic biomarkers, achieving an AUC of 0.94, with 93.8% sensitivity and 91.7% specificity. Plasma cytokine analysis revealed elevated levels of IL-1beta, IL-6, and TNF in individuals with long COVID (10). Functional neuroimaging, particularly [^18^F]-FDG PET, has shown promise in detecting brain alterations associated with cognitive impairment after COVID-19. Characteristic findings such as hypometabolism in anterior brain regions and slowed EEG activity provide objective evidence of neurophysiological changes and may aid in the development of diagnostic tools for long COVID (11). Emerging evidence from transcriptomics, cytokine profiling, and functional neuroimaging offers promising objective biomarkers to improve the diagnosis of long COVID, which currently relies heavily on subjective symptom reporting.

Due to insufficient awareness among healthcare professionals, many long COVID patients are misdiagnosed with anxiety or depression and fail to receive appropriate care (12, 13). The study by Au et al. (12) analyzed experiences from 334 individuals with long COVID and found that many participants reported their symptoms were dismissed or misattributed to mental health conditions. Affected individuals often report being dismissed or misunderstood and consequently turn to social media platforms for support and validation (12, 14, 15). This highlights the urgent need to educate medical professionals, improve symptom recognition, and increase diagnostic accuracy. We must acknowledge that the first step toward effective care of long COVID is acknowledging the legitimacy of patients’ experiences. What’s more, long COVID imposes substantial economic losses and poses major obstacles to return to work. Population-level evidence from longitudinal UK cohorts links self-reported long COVID with increased odds of labor-market inactivity and long-term sickness absence within 6–12 months of infection, contextualizing the observed work disruption (16). A meta-analysis also indicated that 60.9% of post-COVID patients return to work ≥12 weeks after infection, often with modified duties/h, underscoring persistent work limitations despite return-to-work (17).

Another pressing challenge is the lack of effective, evidence-based treatments. Current clinical trials predominantly target isolated symptoms—such as fatigue, cognitive dysfunction, or gastrointestinal issues—without addressing the complex, multi-system nature of long COVID (18, 19). This fragmented approach often fails to provide lasting relief. While there is currently no specific pharmacological therapy for long COVID, several symptomatic treatments have been shown to alleviate disease burden. Furthermore, emerging evidence highlights the potential role of dietary interventions (e.g., anti-inflammatory diets and micronutrient supplementation) (20) and probiotics in modulating gut–lung axis dysfunction and systemic inflammation (21). Pulmonary rehabilitation, encompassing aerobic and resistance training, breathing exercises, and education, has been demonstrated to improve exercise capacity, dyspnea, and quality of life in long COVID patients (22).

In China, many patients seek help through traditional Chinese medicine (TCM). It was reported that TCM possesses the ability to suppress cytokine storms, ameliorate coagulation dysfunction, and reduce myocardial injury, which suggest that TCM may offer therapeutic advantages in addressing hyperinflammatory responses, coagulopathies, and cardiac complications commonly observed during the post-COVID-19 recovery phase (23). The efficacy of Qingjin Yiqi granules in 388 patients with long COVID was evaluated and demonstrated significant improvements in dyspnea and fatigue compared to controls (24). A systematic review and meta-analysis found that receiving TCM treatment is associated with a post-treatment clinical reduction in depression and anxiety in long COVID adults, compared to the control (25). However, TCM treatments rely on individualized syndrome differentiation, which varies significantly across practitioners. This variability limits standardization and reproducibility, posing challenges for integration into guideline-based care.

We call on the medical community to take concrete steps to address long COVID. First, recognize it as a legitimate clinical entity in line with WHO and enhance healthcare provider training through continuing medical education programs, standardized case vignettes, and clinical decision-support tools aiming at improving early recognition and management of long COVID. What’s more, standardized diagnostic criteria and reliable biomarkers should be develop. And, above all, healthcare provider should adopt integrative, patient-centered care models, including multidisciplinary clinics and evidence-supported traditional therapies. By listening to patients and embracing the complexity of long COVID, we can provide more effective care and restore quality of life for millions affected worldwide.

## Data Availability

The original contributions presented in the study are included in the article/supplementary material, further inquiries can be directed to the corresponding authors.
